# Silica-associated systemic lupus erythematosus with lupus nephritis and lupus pneumonitis: A case report and a systematic review of the literature

**DOI:** 10.1097/MD.0000000000028872

**Published:** 2022-02-18

**Authors:** Kazuhiko Fukushima, Haruhito A. Uchida, Yasuko Fuchimoto, Tomoyo Mifune, Mayu Watanabe, Kenji Tsuji, Katsuyuki Tanabe, Masaru Kinomura, Shinji Kitamura, Yosuke Miyamoto, Sae Wada, Taisaku Koyanagi, Hitoshi Sugiyama, Takumi Kishimoto, Jun Wada

**Affiliations:** a Department of Nephrology, Rheumatology, Endocrinology and Metabolism, Okayama University Graduate School of Medicine, Dentistry and Pharmaceutical Sciences, Okayama, Japan; b Department of Chronic Kidney Disease and Cardiovascular Disease, Okayama University Graduate School of Medicine, Dentistry and Pharmaceutical Sciences, Okayama, Japan; c Department of Respiratory Medicine, Okayama Rosai Hospital, Okayama, Japan; d Department of Human Resource Development of Dialysis Therapy for Kidney Disease, Okayama University Graduate School of Medicine, Dentistry and Pharmaceutical Sciences, Okayama, Japan.

**Keywords:** lupus nephritis, lupus pneumonitis, silicosis, SLE

## Abstract

**Introduction:**

Several epidemiological studies have shown that silica exposure triggers the onset of systemic lupus erythematosus (SLE); however, the clinical characteristics of silica-associated SLE have not been well studied.

**Patient concerns:**

A 67-year-old man with silicosis visited a primary hospital because of a fever and cough. His respiratory condition worsened, regardless of antibiotic medication, and he was referred to our hospital.

**Diagnosis:**

The patient showed leukopenia, lymphopenia, serum creatinine elevation with proteinuria and hematuria, decreased serum C3 level, and was positive for anti-double stranded DNA antibody, anti-nuclear antibody, and direct Coombs test. He was diagnosed with SLE. Renal biopsy was performed, and the patient was diagnosed with lupus nephritis (class IV-G(A/C) + V defined by the International Society of Nephrology/Renal Pathology Society classification). Computed tomography revealed acute interstitial pneumonitis, bronchoalveolar lavage fluid showed elevation of the lymphocyte fraction, and he was diagnosed with lupus pneumonitis.

**Interventions:**

Prednisolone (50 mg/day) with intravenous cyclophosphamide (500 mg/body) were initiated.

**Outcomes:**

The patient showed a favorable response to these therapies. He was discharged from our hospital and received outpatient care with prednisolone slowly tapered off. He had cytomegalovirus and herpes zoster virus infections during treatment, which healed with antiviral therapy.

**Review:**

We searched for the literature on sSLE, and selected 11 case reports and 2 population-based studies. The prevalence of SLE manifestations in sSLE patients were comparative to that of general SLE, particularly that of elderly-onset SLE. Our renal biopsy report and previous reports indicate that lupus nephritis of sSLE patients show as various histological patterns as those of general SLE patients. Among the twenty sSLE patients reported in the case articles, three patients developed lupus pneumonitis and two of them died of it. Moreover, two patients died of bacterial pneumonia, one developed aspergillus abscesses, one got pulmonary tuberculosis, and one developed lung cancer.

**Conclusion:**

Close attention is needed, particularly for respiratory system events and infectious diseases, when treating patients with silica-associated SLE using immunosuppressive therapies.

## 1 Introduction

Silica exposure is a known risk factor for systemic lupus erythematosus (SLE) induction. A series of case reports (Table 1) and epidemiological studies^[^1^,^^2^^]^ have been reported for silica-associated SLE (sSLE) from past to present. However, the clinical course and characteristics of sSLE have not yet been well studied. In particular, it is not clear whether there are any differences between sSLE and idiopathic SLE in terms of clinical features. Herein, we report a case of a male patient with silicosis who developed lupus nephritis and acute interstitial pneumonitis (lupus pneumonitis). We also performed a systematic review of the literature on sSLE to examine the clinical characteristics of sSLE, particularly the prevalence of SLE manifestations and the clinical course after treatment.

**Table 1:** The cumulative prevalence of SLE manifestations in ACR criteria with lupus pneumonitis in sSLE patients calculated from 12 case reports and 2 population-based studies.

Author (published year/country)	No.	Age	Sex	Silicosis	Malar rash	Discoid rash	Photo sensitivity	Oral ulcer	Arthritis	Serositis	Renal disorder	Neuro logical disorder	Hemato logical disorder	Immuno logical disorder	ANA	Lupus pneumonitis
<Case report>																
Our case (2021/Japan)	1	57	M	+						+	+		+	+	+	+
Tsuchiya et al (2017/Japan)^[43]^	2	63	M	+						+			+	+	+	
Lucas et al (2014/UK)^[44]^	3	64	M	+					+	+	+		+	+	+	
Yamazaki et al (2007/Japan)^[25]^	4	77	M	+						+	+		+	+	+	
Hrycek (2007/Poland)^[45]^	5	62	M	+					+	+	+			+	+	
Holanda et al (2003/Brazil)^[46]^	6	40	M	+					+	+			+		+	
Costallat et al (2002/Brazil)^[47]^	7	40	M	+					+	+	+			+	+	
	8	61	M	+					+	+			+	+	+	
Rosenman et al (1999/USA)^[48]^	9	61	M	+					+					+	+	
Haustein et al (1998/Germany)^[12]^	10	51	M	+	+	+	+		+		+		+	+	+	
	11	46	M		+		+	+	+	+			+	+	+	
	12	63	M	+	+	+	+	+	+	+			+	+	+	
	13	58	M	+	+		+		+		+	+		+	+	
Ozoran et al (1997/Turkey)^[49]^	14	65	M	+					+				+	+	+	
Koeger et al (1995/France)^[50]^	15	36	M	+	+		+		+	+	+			+; 3-; 1	+	+
	16	53	M	+	+				+	+	+				+	+
	17	55	M	+			+		+	+					+	
	18	43	M		+				+						+	
Bolton et al (1981/USA)^[11]^	19	39	M	+					+		+	+		+	+	
	20	43	M	+					+		+				+	
Total of case reports		54.0^∗^	M20 F0	18/20	7/20 (35%)	2/20 (10%)	6/20 (30%)	2/20 (10%)	17/20 (85%)	13/20 (65%)	11/20 (55%)	2/20 (10%)	10/20 (50%)	17/20 (85%)	20/20 (100%)	3/20 (15%)
<Population-based study>																
Parks et al (2002/USA)^[1]^			M15 F36		23/51 (45%)	9/51 (18%)	22/51 (43%)	10/51 (20%)	39/51 (76%)	21/51 (41%)	13/51 (25%)	1/51 (2%)	^†^	^‡^	45/51 (88%)	
Conrad et al (1996/Germany)^[13]^		52.6^∗^	M28 F0	19/28	19/28 (68%)	13/28 (46%)	11/28 (39%)	3/28 (11%)	16/28 (57%)	13/28 (46%)	10/28 (36%)	2/28 (7%)	24/28 (86%)	12/28∼^§^ (43%)	25/28 (89%)	
Cumulative prevalence (No.10–13 were excluded)			M59 F36		45/95 (47%)	22/95 (23%)	35/95 (37%)	13/95 (14%)	68/95 (72%)	44/95 (46%)	33/95 (35%)	4/95 (4%)	31/44 (70%)		86/95 (91%)	

All of these patients were confirmed to have silicosis or heavily exposed to silica. Gray background shows manifestation developed after treatment. ACR = American College of Rheumatology, ANA = antinuclear antibody, F = female, M = male, SLE = systemic lupus erythematosus, sSLE = silica-associated systemic lupus erythematosus.

∗ Mean age.

† 12 of lymphopenia, 6 of thrombopenia, 5 of leukopenia patients of 51 patients.

‡ 13 of anti dsDNA antibody, 8 of anti Sm antibody, 3 of anti cardiolipin antibody-positive pat1ients of 51 patients.

§ Sera of 10 of 28 patients were not available for autoantibody analysis.

## 2 Case presentation

**Figure 1:**
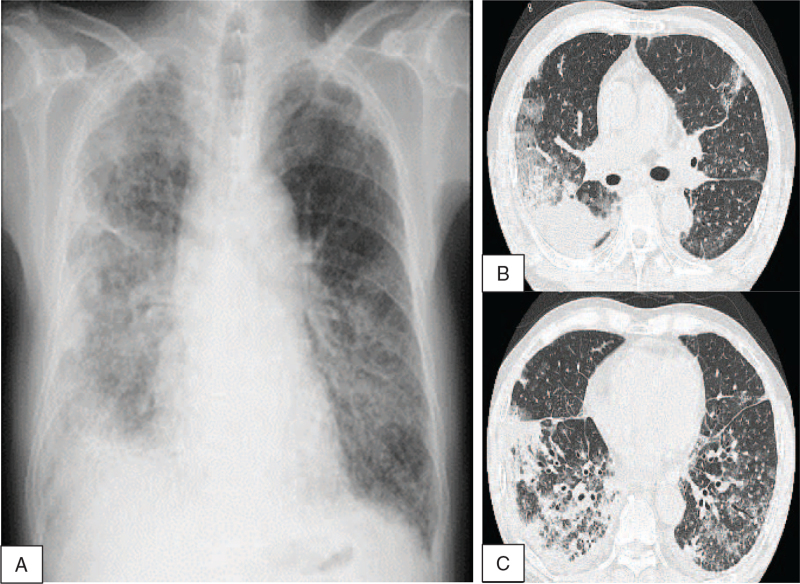
(A) Chest radiography shows consolidation of the bilateral lung fields. The right costophrenic angle is dull. (B and C) Computed tomography of the chest reveals bilateral centrilobular micronodular opacities compatible with silicosis, and it also shows enlarged ground-grass shadows, infiltrative shadows and increased pleural effusion, compatible with acute interstitial pneumonia (lupus pneumonitis) and pleuritis.

**Figure 2:**
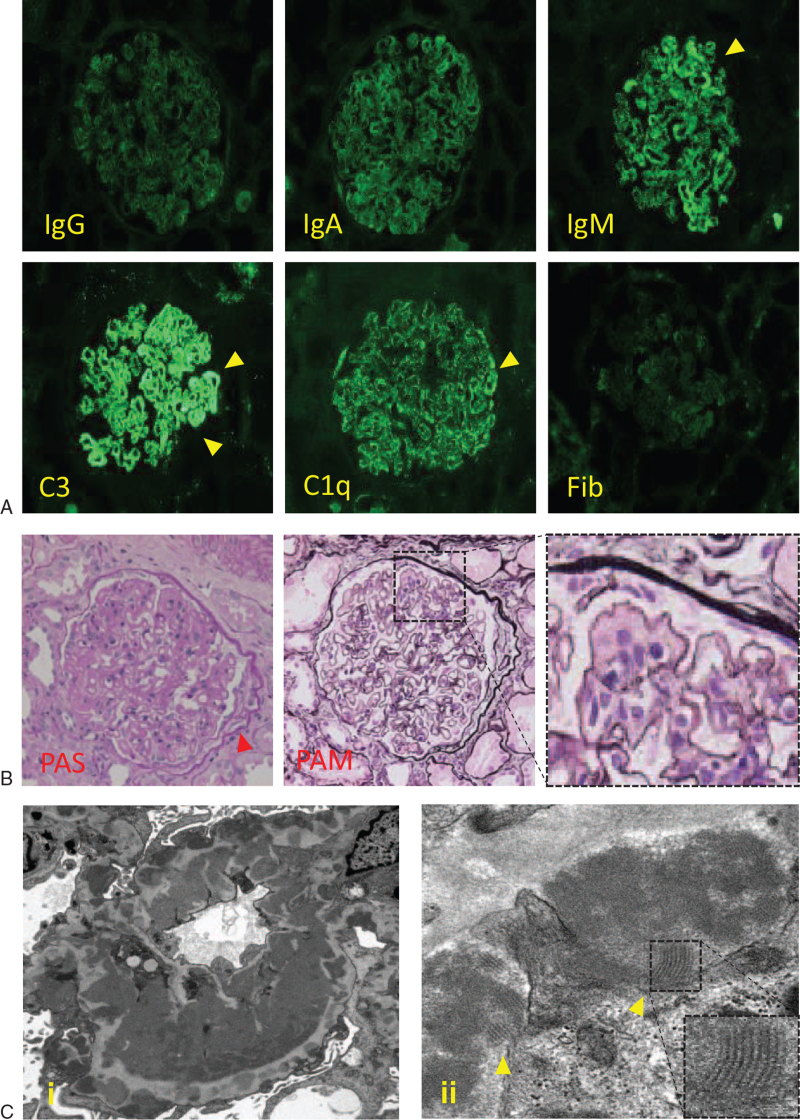
(A) Immunofluorescence microscopy reveals staining on the glomerular capillary walls and partly on mesangial region with all 3 immunoglobulins and complements (C3 and C1q), and weak staining with fibrinogen (×400). In particular, IgM, C3 and C1q depositions showed fringe-like pattern (arrowhead) (×400). (B) Light microscopy reveals diffuse proliferative nephritis (×400). Global wire loop lesions, endo-capillary proliferation, and fibrocellular crescent (arrowhead) are observed with Periodic Acid-Schiff staining. (C) Electron microscopy reveals diffuse and massive sub-endothelial dense deposits, intramembranous deposits and sub-epithelial deposits (i; ×5000). Some of those deposits contained fingerprint-like structures (arrowhead) (ii; ×30,000).

A 67-year-old man, who had worked in a quarry for decades and was diagnosed with silicosis at age of 54 years, visited a primary hospital due to fever and cough. The chest radiography revealed a new ground-glass shadow in the right middle and under lung fields, and the SpO_2_ was 91%. The patient was diagnosed with bacterial pneumonia, and antibiotics were administered. Since his fever and cough persisted for 9 days, he was admitted to another hospital. Laboratory tests demonstrated an elevated serum creatinine (Cr) concentration of 2.87 mg/dL, which was 1.39 mg/dL a year before, and gradually increased thereafter. Laboratory tests also showed leukopenia (1.71 × 10^3^ cells/μL) with lymphopenia (100 cells/μL), anemia (hemoglobin 9.7 g/dL), thrombocytopenia (115 × 10^3^ cells/μL), elevated serum levels of anti-nuclear antibody (320×, homogeneous and speckled pattern), anti-double stranded DNA (dsDNA) antibody (99.2 IU/mL), and decreased serum level of C3 (31.1 mg/dL). A direct Coombs test result was positive, but the serum haptoglobin level did not decrease (315 mg/dL). Urine analysis revealed proteinuria (2.27 g/g Cr) and microscopic hematuria with dysmorphic red blood cells (RBCs) (30–49 /high-power field) and granular cylinders. Computed tomography (CT) of the chest revealed ground-glass shadows and infiltrative shadows in the right middle and under the lung lobes with right pleural effusion. Bronchoalveolar lavage fluid showed total cell count of 15.0 × 10^6^, in which the lymphocyte fraction was elevated (39.5% of lymphocytes, 1.0% of neutrophils, and 58.5% of macrophages). Ten days after admission, his respiratory condition worsened and he was referred to our hospital. On the first day of admission, his temperature was 38.0°C and SpO_2_ was 90% under 3 L oxygen inhalation on nasal cannula. His serum Cr concentration was elevated to 3.20 mg/dL. Chest radiography showed consolidation of the bilateral lung fields, the right costophrenic angle was dull (Fig. 1A), and chest CT showed expanded ground-glass shadows, infiltrative shadows, and increased pleural effusion (Figure 1B, C). Based on these observations, he was finally diagnosed with SLE with rapidly progressive glomerulonephritis and acute interstitial pneumonitis based on the criteria defined by the American College of Rheumatology (ACR)^[^3^,^^4^^]^ and Systemic Lupus International Collaborating Clinics (SLICC)^[5]^: leukopenia, lymphopenia, renal disorder, serositis, anti-dsDNA antibody, anti-nuclear antibody, low C3, and direct Coombs test. Glucocorticoid therapy was initiated on the second day after admission (prednisolone, 50 mg/day for 15 days, after which the dosage was gradually tapered). Renal biopsy was conducted 10 days after admission; IgG, IgA, IgM, C3, and C1q granular depositions were observed on the capillary walls and partly in the mesangial region by immunofluorescence (Fig. 2A). In particular, IgM, C3, and C1q depositions showed fringe-like patterns (Fig. 2A). Fifteen glomeruli were confirmed by light microscopy, and 2 showed global sclerosis. Diffuse global wire loop lesions and endocapillary proliferation were observed with periodic acid-Schiff and periodic acid-methenamine silver staining (Fig. 2B). Fibrocellular crescents were also found in 2 of 15 glomeruli (Fig. 2B). Under electron microscopy, subepithelial, intramembrane, subendothelial, and paramesangial dense deposits were observed, some of which contained fingerprint-like structures (Fig. 2C). These findings were consistent with lupus nephritis class IV-G(A/C) + V, active and chronic lesions of diffuse global proliferative and sclerosing lupus nephritis, and membranous lupus nephritis, as defined by the International Society of Nephrology/Renal Pathology Society (ISN/RPS) classification.^[^6^–^8^]^ Based on this pathological diagnosis, intravenous cyclophosphamide therapy (500 mg/body weight) was administered 24 and 53 days after admission. The patient's fever and respiratory condition gradually improved after the initiation of treatment, and the oxygen flow rate decreased to 1 L/min on the nasal cannula. Chest CT on 32 days after admission revealed diminishment of ground-glass shadows and infiltrative shadows in the lung and reduction of pleural effusion. On 41 days after admission, the cytomegalovirus antigenemia test was positive (10/50,000 cells) without any symptoms. Intravenous ganciclovir (2.5 mg/kg) was administered for 14 days, after which the cytomegalovirus antigenaemia test was negative. Laboratory tests on the 59 days after admission showed reduction of serum Cr concentration to 1.29 mg/dL and improvement of pancytopenia. In the urine analysis, proteinuria was reduced to 1.46 g/g Cr with RBCs 5-9/high-power field, and the granular cylinders disappeared. The patient remained asymptomatic under 1 L oxygen inhalation only when walking and was discharged from our hospital 62 days after admission. He had herpes zoster in his right abdominal skin 19 days after discharge from our hospital; therefore, the third intravenous cyclophosphamide therapy was discontinued. Although the serum Cr level was elevated to 1.45 mg/dL due to dehydration at that time, pancytopenia did not worsen (leukocyte 7.00 × 10^3^ cells/μL, hemoglobin 10.3 g/dL, thrombocyte 18.2 × 10^3^ cells/μL), proteinuria was reduced to 0.71 g/g Cr, and urinary RBCs disappeared. Chest CT showed further improvement in lung shadows and pleural effusion. His herpes zoster healed after amenamevir initiation, and he received outpatient care with slowly tapered prednisolone.

## 3 Systematic review

### 3.1 Methods

We searched PubMed for original articles on sSLE, published between 1955 and November 2021. On PubMed (searched on January 1st, 2022), we performed the following literature search: ('systemic lupus erythematosus’ OR 'SLE’ OR ’lupus’) AND ('silica’ OR 'silicosis’). The bibliographic search was also extended using the links ‘related articles of PubMed and the reference lists of some key studies. The final selection of the included articles involved careful reading and analysis of the entire text. The entire process followed during the systematic review is shown in the Supplementary Figure. The flow chart is based on the PRISMA flow diagram, which was proposed based on the official preferred reporting items for systematic reviews and meta-analysis. The bibliographic search yielded 262 articles in PubMed, and 2 additional records were identified through other sources.

Based on the title, we eliminated 145 articles that were not relevant to SLE and silica exposure. Articles on basic research and specific molecules or testing were eliminated. After the abstract revision, we eliminated 78 articles that were not relevant to patients with SLE who suffered from silicosis or had been exposed to silica. After reading the full text, we excluded 8 review articles, 11 articles that lacked a description of SLE manifestations, 5 articles that were not available, 2 articles that were not written in English, 1 article in which case anti-neutrophil cytoplasmic antibody (ANCA)-associated vasculitis was complicated,^[9]^ and 1 article^[10]^ in which the patients were considered to duplicate with the other article written by the same authors.^[11]^

Finally, we selected 11 case reports and 2 population-based studies on sSLE patients (Table 1). We then extracted data on SLE manifestations in patients with sSLE. Most of these articles were published before the SLICC criteria for SLE classification were established in 2012,^[5]^ and SLE manifestations in the articles were described on the basis of ACR criteria (established in 1982 and revised in 1997).^[^3^,^^4^^]^ Accordingly, we adopted items in the ACR criteria to compare the prevalence of SLE symptoms between patients with sSLE and general SLE. We confirmed that we did not adopt the SLE case that called for lupus erythematosus cell preparation test for the diagnosis of SLE, which was excluded from the ACR criteria in the 1997 revision.^[3]^ We adopted case reports of sSLE published before 1982, if the symptoms of the patients in the articles could be confirmed to match the ACR criteria in 1997 or the SLICC criteria. Thus, we collected 20 sSLE patients from 12 case reports, including our case, and 79 sSLE patients from 2 population-based studies (Table 1). All patients were confirmed to have silicosis or were heavily exposed to silica. These patients were followed-up over years or decades by medical institutions from the diagnosis of silicosis or having been exposed to silica to the diagnosis of SLE, and were checked after treatment initiation; therefore, we calculated the cumulative prevalence of SLE manifestations from the description of their articles. We could not exclude the possibility that the patients in a case report by Haustein et al^[12]^ might be included in a population-based study by Conrad et al,^[13]^ as both of their studies were reported from Germany in the 1990s (Table 1). To avoid overestimation by double counting of the same patients, we assumed that all 4 patients reported by Haustein et al^[12]^ were included in the study by Conrad et al^[13]^ and excluded when calculating the prevalence of each SLE symptom (we confirmed that our conclusion described below did not change if we included those 4 patients in the calculation). We confirmed that there was no other overlapping of patients among the 12 case reports and 2 population-based studies based on when and where the articles were published, description of clinical course, or age of patients in the articles. We could not grasp the details of hematological disorders (hemolytic anemia, leukopenia, lymphopenia, or thrombocytopenia) and immunological disorders (anti-DNA antibody, anti-Sm antibody, or anti-phospholipid antibody); therefore, we calculated the total prevalence of hematological disorders and immunological disorders. Parks et al^[1]^ did not report the total number of hematological and immunological disorders; thus, we excluded their data when calculating the cumulative prevalence of hematological and immunological disorders.

## 4 Results

Table 1 shows the cumulative prevalence of SLE manifestations in the ACR criteria together with lupus pneumonitis in patients with sSLE. We could not calculate the prevalence of immunological disorders because its prevalence by Conrad et al (43%)^[13]^ was obviously lower than would be expected in general SLE patients (70%–80% for anti-dsDNA antibody alone),^[14]^ which would mainly be due to the lack of data. Conrad et al stated in their article that sera of 10 of 28 patients were not available for autoantibody analysis.^[13]^ The prevalence of immunological disorders in case report collection (85%) was more comparable to that of general SLE.^[14]^ The prevalence of SLE manifestations in sSLE patients was within the range of general SLE patients reported so far,^[^15^–^18^]^ although the reported prevalence of SLE manifestations is wide-ranging and varies depending on the population group studied. The prevalence of neurological disorders in this study (4%) was more comparable to that of elderly-onset SLE than early-onset SLE,^[19]^ which was reported to occur less frequently in elderly SLE patients than in younger SLE patients in many studies.^[^20^–^24^]^ Lupus pneumonitis was reported to develop in 3 of 20 patients in case report collection (15%).

Table 2 shows the clinical course of the patients with sSLE in the case report collection. Twelve out of 20 patients (60%) showed a favorable response to initial immunosuppressive therapies. However, 7 patients (35%) had fatal events during follow-up; 2 patients died of bacterial pneumonia (cases 6 and 19), 1 died of lupus pneumonitis (case 15), 1 died of Aspergillus abscesses with lupus pneumonitis (case 16), 1 had pulmonary tuberculosis (case 7), 1 developed lung cancer with liver metastasis (case 5), and 1 died of unknown cause (case 17). Regarding the clinical course of lupus pneumonitis, Case 1 (our patient) and Case 15 showed favorable responses to the initial therapies, although in the latter case, a fatal relapse occurred after accidental withdrawal of prednisolone. On the contrary, case 16 developed lupus pneumonitis together with Aspergillus abscesses (as mentioned above) and died from hypoxemia due to pneumothorax following necrosis of the opacities.

**Table 2:** Treatment course and/or outcome of silica-associated SLE patients in the case reports collection.

Author (published year/country)	No.	Age/sex	Therapy	Treatment course/outcome
Our case (2021/Japan)	1	57/M	PSL, IVCY	Favorable response was shown, but **cytomegalovirus and herpes zoster virus infections occurred.**
Tsuchiya et al (2017/Japan)^[43]^	2	63/M	PSL	Favorable response
Lucas et al (2014/UK)^[44]^	3	64/M	PSL, AZP	Favorable response
Yamazaki et al (2007/Japan)^[25]^	4	77/M	mPSL, PSL	Favorable response
Hrycek (2007/Poland)^[45]^	5	62/M	PSL	Clinical remission was achieved, but after several years, but **lung cancer with liver metastasis developed**.
Holanda et al (2003/Brazil)^[46]^	6	40/M	Corticosteroid	Favorable response was shown, but after 8months **respiratory infection with ARDS occurred and he died.**
Costallat et al (2002/Brazil)^[47]^	7	40/M	PSL, IVCY, AZP	Favorable response was shown. **Pulmonary tuberculosis developed after that**, but multidrug therapy worked.
	8	61/M	PSL	favorable response
Rosenman et al (1999/USA)^[48]^	9	61/M	Corticosteroid HCQ	(No mention)
Haustein et al (1998/Germany)^[12]^	10	51/M	mPSL, CY	(No mention)
	11	46/M	mPSL, AZP	(No mention)
	12	63/M	mPSL, MTX	(No mention)
	13	58/M	mPSL, AZP	(No mention)
Ozoran et al (1997/Turkey)^[49]^	14	65/M	(no mention)	(No mention)
Koeger et al (1995/France)^[50]^	15	36/M	PSL	Favorable response was shown, but **accidental withdrawal caused a fatal relapse, and he died of acute respiratory failure.**
	16	53/M	PSL	After 1 year of PSL therapy, **he developed lupus pneumonitis, aspergillus abscesses, pneumothorax, and he died.**
	17	55/M		He refused to receive treatment. **He died suddenly** after 9 years of a benign course.
	18	43/M	PSL	Remission was achieved. **He suffered from relapse** but became disease-free after that without therapy.
Bolton et al (1981/USA)^[11]^	19	39/M	PSL	Favorable response was shown, but **gram negative pneumonia, lupus cerebritis, intracranial hematoma developed and he died**.
	20	43/M	mPSL, PSL	favorable response

Bold font shows adverse events or poor prognosis. ARDS = acute respiratory distress syndrome, AZP = azathioprine, CY = cyclophosphamide, HCQ = hydroxychloroquine, IVCY = intravenous cyclophosphamide, M = male, mPSL = methyl prednisolone, MTX = methotrexate, PSL = prednisolone, SLE = systemic lupus erythematosus.

Table 3 shows the renal histological findings of the patients with sSLE who underwent renal biopsy. In addition to our case, there were at least 2 other cases that were considered to be lupus nephritis. Case 4 by Yamazaki et al^[25]^ was confirmed to be consistent with lupus nephritis class III (A/C) of the ISN/RPS classification. Case 20 by Bolton et al in 1981^[11]^ would be considered as class IV + V lupus nephritis according to the histological descriptions of focal glomerular hypercellularity, crescents in 50% of glomeruli, and global subepithelial electron dense deposits.

**Table 3:** The renal histological findings of silica-associated SLE patients who underwent renal biopsy.

Author (published year/country)	No.	Age/sex	Pathological findings
Our case (2021/Japan)	1	67/M	Lupus Nephritis IV (A/C)+V
Yamazaki et al (2007)^[25]^	4	77/M	Lupus Nephritis III (A/C)
Koeger et al (1995/France)^[50]^	15	36/M	“Segmental and focal glomerulonephritis”
	16	53/M	“Diffuse proliferative glomerulonephritis ”
Cledes et al (1983)^[51]^	#	59/M	“Focal glomerulonephritis with IgG, IgA, C1q deposition”
Bolton et al (1981/USA)^[11]^	19	39/M	Focal glomerular hyper cellularity, sclerosis, adhesions, tubular necrosis with crescents in 15% of glomeruli
			<EM> Electron dense GBM deposits (focal)
			<IF> Deposit; IgG (focal, GBM)
	20	43/M	Focal glomerular hyper cellularity and sclerosis
			Adhesions, tubular necrosis with crescents in 50% of glomeruli
			Interstitial infiltrate (lymphocyte/plasma cell)
			<EM> Electron dense GBM deposits (extensive) GBM reduplication, microtubules
			<IF> Deposit; IgG (GBM), IgM, C3 (GBM, Mes), C1q (GBM, Mes)

#, Abstract only was available, EM = electron microscope, GBM = glomerular basement membrane, IF = immunofluorescence, M = male, Mes = mesangium, SLE = systemic lupus erythematosus.

## 5 Discussion

Several epidemiological studies have shown that silica exposure triggers the onset of autoimmune diseases such as rheumatoid arthritis, ANCA-associated vasculitis, systemic sclerosis, and SLE.^[26]^ Phagocytosis of crystalline silica by alveolar macrophages causes chronic inflammation via lysosomal rupture followed by caspase-1 activation and an increase in IL-1α secretion followed by nuclear factor-kappa B transcription, which underlies the onset of autoimmune diseases.^[27]^ In addition, experimental long-term exposure to crystalline silica has been reported to cause regulatory T cell suppression and autoimmunity activation.^[28]^ Among these autoimmune diseases, SLE has been well-studied for its association with silica exposure and/or silicosis. Parks et al reported in their population-based study that occupational silica exposure increased the odds ratio of SLE development.^[1]^ Morotti et al also reported a possible association between occupational exposure to crystalline silica and SLE, particularly in patients with silicosis, in their meta-analysis of epidemiological study.^[2]^ There have been some differences in the clinical characteristics of silica-associated SLE (sSLE) and idiopathic SLE. Although general SLE occurs mainly in young women,^[^29^,^^30^^]^ sSLE patients reported to date are predominantly middle-aged and older men. Conrad et al identified 28 SLE patients in a cohort of 15,000 heavily silica-exposed uranium miners, all of whom were male, and the age of SLE onset was between 40 and 63 years (on average 52.6).^[13]^ In this study, the latency between the beginning of exposure and disease manifestation was 11 to 50 years (on average 30.3),^[13]^ which would explain the older age of the sSLE patients. Male dominance in the number of sSLE patients reported may be due to the fact that men tend to have a silica-exposing job more than women, such as quarries and miners. Women who are heavily exposed to silica have been reported to develop sSLE, similar to men, in a population-based study.^[1]^ Whether there are differences in the prevalence of SLE manifestations between sSLE and general SLE is controversial. One population-based study reported that sSLE patients had a decreased frequency of arthritis and photosensitivity compared with general SLE patients.^[13]^ In contrast, Parks et al reported that hemolytic anemia and leukopenia were less common in sSLE patients than in general SLE.^[1]^ Additionally, it is unclear whether there are any differences in the clinical characteristics between sSLE and general SLE.

The cumulative prevalence of SLE manifestations in sSLE patients was within the range reported to date in general SLE patients.^[^15^–^18^]^ This result might be natural because the reported prevalence of each SLE manifestation is wide-ranging. For example, the neurological disorders in ACR criteria are seizures and psychosis, and their reported prevalence in general SLE is 2% to 20% and 5% to 52%, respectively.^[15]^ The prevalence of neurological disorders (as a total of seizures and psychosis) in this analysis was 4%, which is almost below the lower limit of those previously reported.^[15]^ One of the reasons would be the high average age of the sSLE patients; The neurological manifestations of SLE, particularly seizure and psychosis, are reported as more common in children than in adults.^[^20^–^24^]^ Aleksandra et al reported that the prevalence of neuropsychiatric manifestations of late-onset SLE (6.6%) is significantly lower than that of early-onset SLE (36.6%),^[19]^ and the former is closer to ours. Interestingly, they also reported that late-onset SLE had significantly lower prevalence than early-onset SLE of malar rash (40.0% vs 73.3%) and photosensitivity (36.7% vs 76.6%),^[19]^ and their prevalence of them in our study was closer to that of late-onset SLE (Table 1). We cannot conclude that sSLE patients are at a lower risk for these manifestations because of the study limitations, and we should note that the difference in the prevalence of manifestations between early onset and elderly onset SLE remains controversial. However, our study indicates that sSLE patients have the onset risk of SLE manifestations at least as well as general SLE patients, particularly as elderly-onset SLE patients.

Lupus pneumonitis is an acute interstitial pneumonia that occurs in 1% to 4% of SLE patients.^[31]^ It presents as fever, cough, dyspnea, or, rarely, bloody sputum.^[^31^,^^32^^]^ Pulmonary infiltration is the major radiographic manifestation, and it appears as a ground-glass shadow or honeycomb appearance on CT scan.^[^33^,^^34^^]^ Histological characteristics include alveolar wall damage and necrosis, inflammatory cell infiltration, edema, hemorrhage, and hyaline membranes.^[31]^ The mainstay treatment for lupus pneumonitis is high-dose corticosteroids (prednisone 1.0–1.5 mg/kg/day),^[35]^ but several cases are resistant to steroids, and in such cases, cyclophosphamide^[36]^ or plasmapheresis^[37]^ are applied. The mortality rate of lupus pneumonitis is reported as 40%,^[38]^ and it could develop diffuse alveolar damage resulting in acute respiratory distress syndrome.^[39]^ These features are almost consistent with our study results; of 3 patients with lupus pneumonitis, 2 showed a favorable response to immunosuppressive therapies, but 2 died from it. Notably, a considerable number of serious respiratory events occurred in addition to lupus pneumonitis in the case report collection: bacterial pneumonia, Aspergillus abscesses, pulmonary tuberculosis, and lung cancer (Table 2). These results are consistent with the fact that silicosis patients are at a high risk of lung cancer and infectious diseases, particularly mycobacterial infections,^[^40^–^42^]^ and the risk would be further increased by immunosuppressive therapy of SLE, or by SLE itself. These findings indicate that respiratory events are among the most important prognostic factors in patients with sSLE.

Renal involvement is considered as one of the most important prognostic factors of SLE and occurs in 34% to 73% of the patients with SLE.^[15]^ Considering that silicosis patients are at higher risk for developing autoimmune diseases which damage kidney besides SLE, such as ANCA-associated vasculitis and systemic sclerosis,^[26]^ urine analysis is important in the regular checkup of silicosis patients. Lupus nephritis is a type of glomerulonephritis secondary to autoimmune disorders in SLE. Lupus nephritis is classified by the ISN/RPS based on histological findings of renal biopsy specimens,^[^6^–^8^]^ and this classification is important in determining the therapeutic strategy for lupus nephritis. As shown in Table 3, reports of lupus nephritis in sSLE patients are limited, and we could not determine some of those cases as lupus nephritis based on their histological descriptions, especially those that were reported before the ISN/RPS classification was established. Case 4 by Yamazaki et al^[25]^ was confirmed to be consistent with class III lupus nephritis. Case 20 by Bolton et al^[11]^ would be class IV + V lupus nephritis according to the histological descriptions, although it was previously reported that the ISN/RPS classification was established. Our case of lupus nephritis matched the histological findings of the class IV + V ISN/RPS classification, and this is the first case report to confirm the fingerprint-like structure in electron-dense deposits. These findings indicate that lupus nephritis in patients with sSLE shows various histological patterns similar to those in general SLE patients. Further case studies are needed to elucidate whether there are any differences in the histological or clinical characteristics of lupus nephritis between sSLE and general SLE patients.

This study has at least 2 limitations that could lead to underestimation of the prevalence of SLE manifestations or fatal events. First, articles published before 1997 did not adopt the anticardiolipin antibody or lupus anticoagulant as the immunological disorder of SLE, which was added to the ACR criteria since 1997.^[3]^ Second, this analysis did not fully grasp the length of the observation period, although these patients were followed-up as mentioned above, which would also underestimate the prevalence of fatal events or SLE manifestations. Further studies are required to clarify the clinical characteristics of sSLE.

## 6 Conclusions

Here, we report a case of SLE in a patient who developed lupus pneumonitis and lupus nephritis during the clinical course of silicosis. Close attention is needed to assess respiratory system events, such as bacterial pneumonia, tuberculosis, and lung cancer, when treating sSLE patients with immunosuppressive therapies, which are highly prevalent in patients with silicosis. Further studies are needed to more accurately reveal the clinical course and characteristics of SLE patients with silicosis.

## Author contributions

**Conceptualization:** Kazuhiko Fukushima.

**Data curation:** Kazuhiko Fukushima, Haruhito A. Uchida, Yasuko Fuchimoto, Tomoyo Mifune, Mayu Watanabe, Kenji Tsuji, Katsuyuki Tanabe, Masaru Kinomura, Shinji Kitamura, Yosuke Miyamoto, Sae Wada, Taisaku Koyanagi, Hitoshi Sugiyama, Takumi Kishimoto, Jun Wada.

**Formal analysis:** Kazuhiko Fukushima.

**Investigation:** Kazuhiko Fukushima, Haruhito A. Uchida, Tomoyo Mifune, Mayu Watanabe, Katsuyuki Tanabe, Masaru Kinomura, Shinji Kitamura, Hitoshi Sugiyama, Jun Wada.

**Methodology:** Kazuhiko Fukushima.

**Supervision:** Haruhito A. Uchida, Takumi Kishimoto, Jun Wada.

**Writing – original draft:** Kazuhiko Fukushima, Haruhito A. Uchida.

**Writing – review & editing:** Haruhito A. Uchida, Yasuko Fuchimoto, Tomoyo Mifune, Mayu Watanabe, Kenji Tsuji, Katsuyuki Tanabe, Masaru Kinomura, Shinji Kitamura, Hitoshi Sugiyama, Takumi Kishimoto, Jun Wada.

## Supplementary Material

Supplemental Digital Content
