# Supplementary material for: Silica-associated systemic lupus erythematosus with lupus nephritis and lupus pneumonitis: A case report and a systematic review of the literature
Source: Medicine (Baltimore). 2022 Feb 18;101(7):e28872. doi: 10.1097/MD.0000000000028872 (PMC9282083; doi:10.1097/MD.0000000000028872)
Supplement: Supplemental Digital Content [file medi-101-e28872-s001.doc]

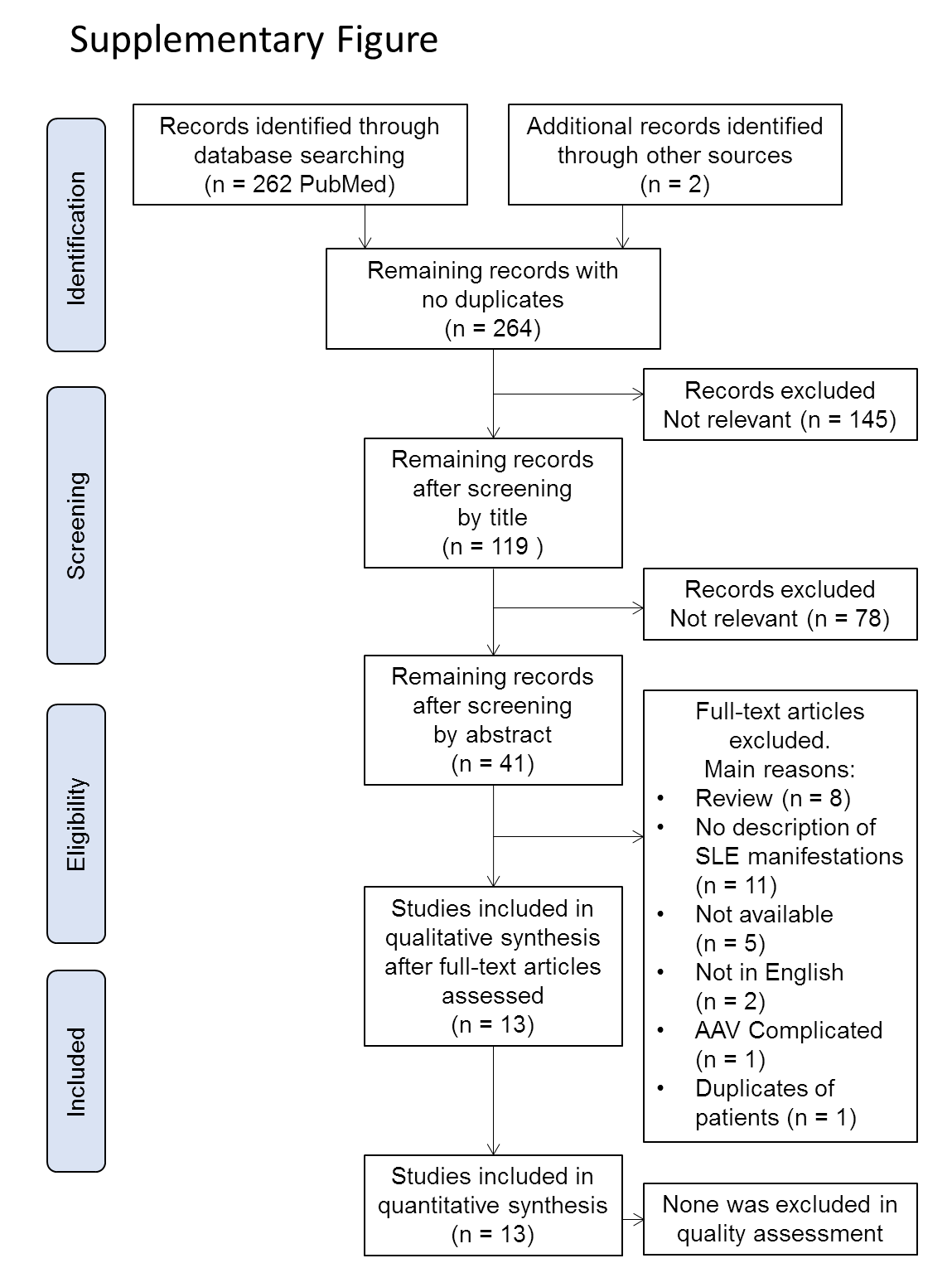
**Supplementary Figure**

Flow chart of search and selection of studies included in the systematic review (AAV: ANCA-associated vasculitis)
